# Supplementary figures and images for: Probabilistic Mixture Models Improve Calibration of Panel-derived Tumor Mutational Burden in the Context of both Tumor-normal and Tumor-only Sequencing
Source: Cancer Res Commun. 2023 Mar 28;3(3):501–9. doi: 10.1158/2767-9764.CRC-22-0339 (PMC10044680; doi:10.1158/2767-9764.CRC-22-0339)

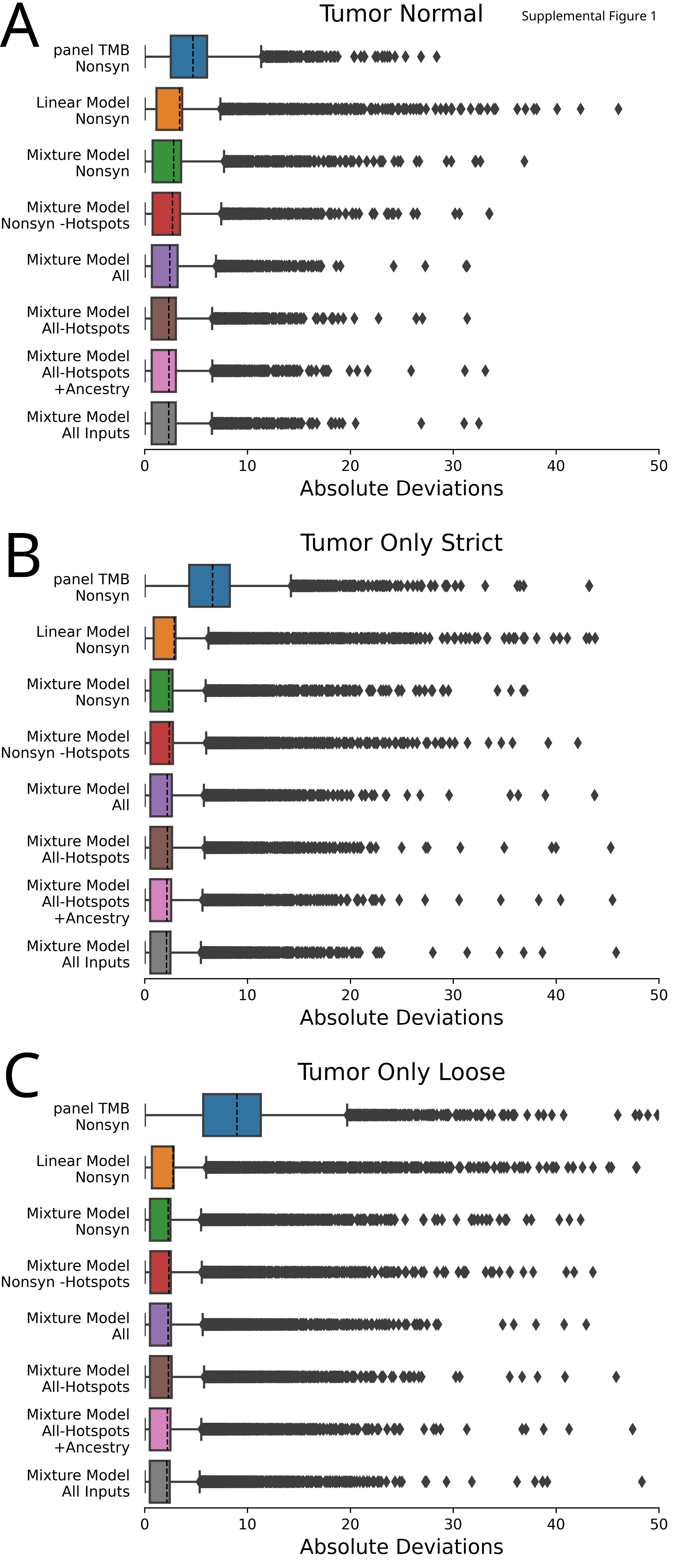

Supplement: Supplemental Figure S1. Box plots of the data represented in Table 1. — Mean of the deviations represented as a dashed line. [file crc-22-0339-s02.png]

**A**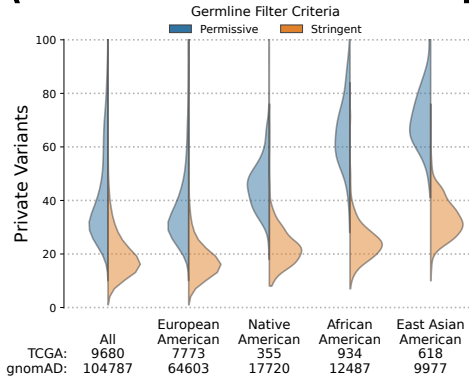**B**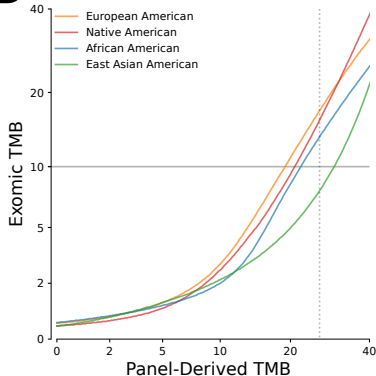**C**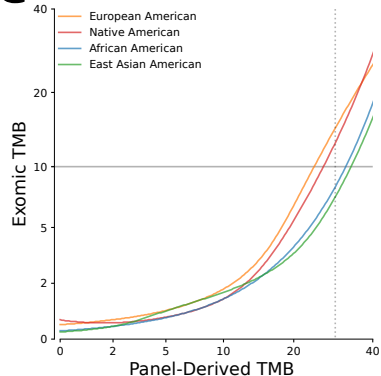

Supplement: Supplemental Figure S2. Effect of ancestry on germline filtering and model fits. — (A) Numbers of germline variants which passed the different filtering criteria for different ancestries. (B) Model fits for models trained on the different cohorts for stringent tumor-only data. (C) Model fits for models trained on the different cohorts for permissive tumor-only data. [file crc-22-0339-s03.pdf]

Supplemental Figure 3

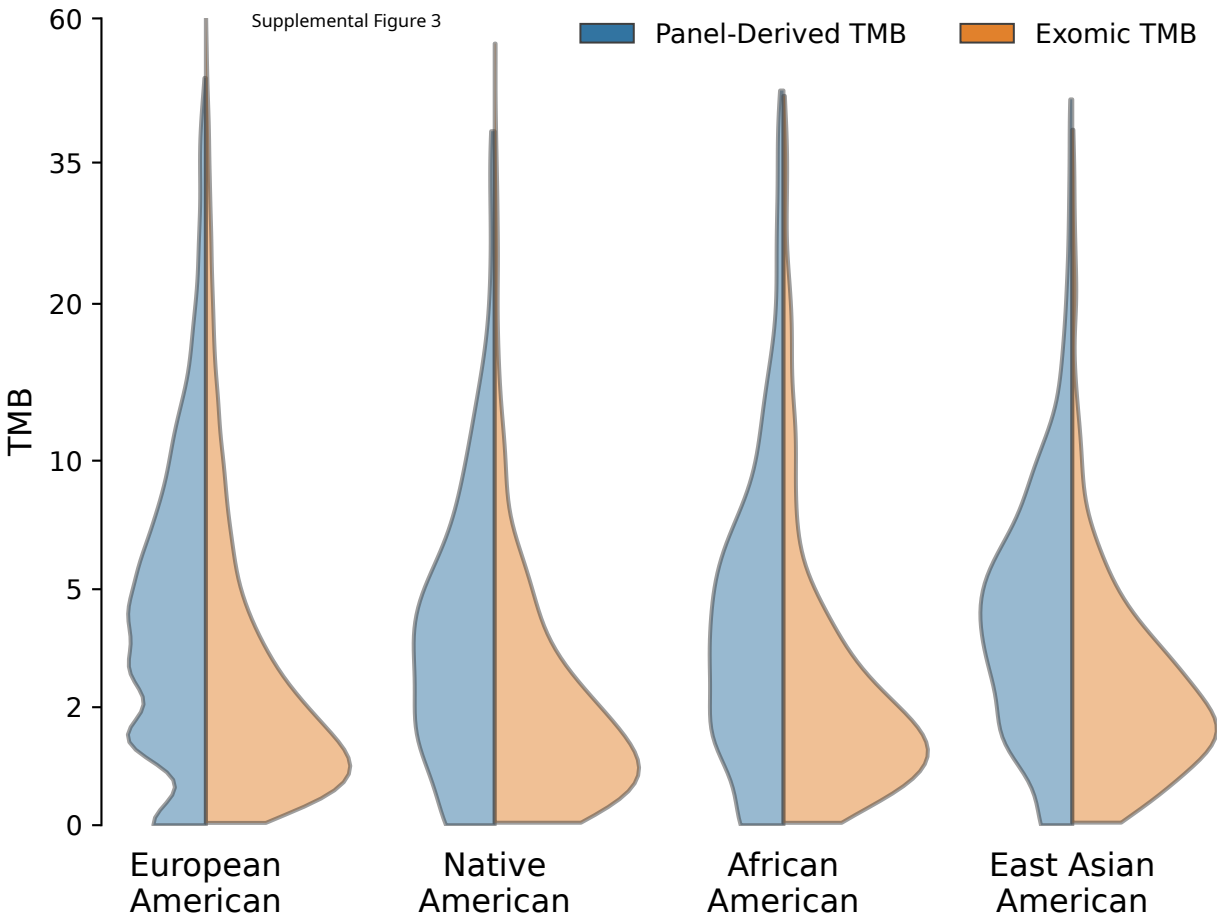

Supplement: Supplemental Figure S3. TMB distributions of different ancestries. — Tumor-normal TMB distributions for panel and exome. [file crc-22-0339-s04.pdf]
