# Supplementary material for: Probabilistic Mixture Models Improve Calibration of Panel-derived Tumor Mutational Burden in the Context of both Tumor-normal and Tumor-only Sequencing
Source: Cancer Res Commun. 2023 Mar 28;3(3):501–9. doi: 10.1158/2767-9764.CRC-22-0339 (PMC10044680; doi:10.1158/2767-9764.CRC-22-0339)
Supplement: Supplemental Table ST1. Detection rates of somatic, exomic TMB > 10 (t) based on stratification by panel-derived TMB measures. — The detection rate of the observed true somatic, exomic TMB being greater than the threshold (t) of 10 based on the various panel derived measures as designated. The linear model and mixture model values are derived from the data in Figure 1 of the manuscript. ŷ: model prediction [file crc-22-0339-s06.pdf]

		Tumor Normal		Tumor Only	
		Exomic TMB $> t$	n	Exomic TMB $> t$	n
Panel TMB	$\hat{y} > t$	53.5%	1486	30.6%	2758
	$\hat{y} \leq t$	1.1%	8201	0.5%	6838
Linear Model	$\hat{y} > t$	94.3%	507	93.1%	379
	$\hat{y} \leq t$	4.4%	9180	5.7%	9217
	$>$ 95% certain, $\hat{y} > t$	100.0%	102	0.0%	0
	$>$ 95% certain, $\hat{y} < t$	0.5%	7733	0.6%	7075
	$<$ 95% certain, $\hat{y} \neq t$	40.1%	1852	33.3%	2521
Mixture Model	$\hat{y} > t$	87.7%	661	81.5%	653
	$\hat{y} \leq t$	3.3%	9026	3.9%	8943
	$>$ 95% certain, $\hat{y} > t$	99.6%	247	94.7%	171
	$>$ 95% certain, $\hat{y} < t$	0.5%	7716	0.8%	7415
	$<$ 95% certain, $\hat{y} \neq t$	34.8%	1724	32.5%	2010
