# Supplementary material for: Probabilistic Mixture Models Improve Calibration of Panel-derived Tumor Mutational Burden in the Context of both Tumor-normal and Tumor-only Sequencing
Source: Cancer Res Commun. 2023 Mar 28;3(3):501–9. doi: 10.1158/2767-9764.CRC-22-0339 (PMC10044680; doi:10.1158/2767-9764.CRC-22-0339)
Supplement: Supplemental Table ST2. TMB regression metrics for different ancestries. — All metrics are from cross validation with counts of all mutations, hotspots, and nonsynonymous mutations as the input. MAE: mean absolute error. MAE and Spearman rank-order correlation are calculated for samples with panel TMB greater than or equal to 5. [file crc-22-0339-s07.docx]

| Data | Ancestry | With Ancestry | MAE | Spearman’s rho |
| --- | --- | --- | --- | --- |
| Tumor Normal | European American | - | 2.45 | 0.81 |
| (matched germline substracted) |  | + | 2.40 | 0.82 |
|  | Native American | - | 1.73 | 0.83 |
|  |  | + | 1.84 | 0.79 |
|  | African American | - | 2.14 | 0.78 |
|  |  | + | 2.17 | 0.78 |
|  | East Asian American | - | 1.87 | 0.68 |
|  |  | + | 1.95 | 0.67 |
| Tumor Only | European American | - | 2.45 | 0.67 |
| (stringent germline filtering) |  | + | 2.27 | 0.67 |
|  | Native American | - | 1.69 | 0.62 |
|  |  | + | 1.68 | 0.61 |
|  | African American | - | 1.88 | 0.59 |
|  |  | + | 1.88 | 0.59 |
|  | East Asian American | - | 1.62 | 0.56 |
|  |  | + | 1.57 | 0.55 |
| Tumor Only | European American | - | 2.50 | 0.58 |
| (permissive germline filtering) |  | + | 2.30 | 0.59 |
|  | Native American | - | 1.73 | 0.52 |
|  |  | + | 1.71 | 0.51 |
|  | African American | - | 1.82 | 0.56 |
|  |  | + | 1.81 | 0.56 |
|  | East Asian American | - | 1.66 | 0.49 |
|  |  | + | 1.54 | 0.50 |
